# Peer-to-Peer Case Review as a Strategy to Improve Sepsis Education in Graduate Medical Education

**DOI:** 10.51894/001c.159860

**Published:** 2026-04-08

**Authors:** Obert Xu, Bryanna De Lima, Kenneth DeVane, David Jones, Mohamud Daya, Christopher Greenlee, Andrew Terrell, Scott Sherry, Haley Manella

**Affiliations:** 1 Emergency Medicine Oregon Health & Science University, Oregon, Portland, OR

**Keywords:** Sepsis education, Graduate medical education, peer-to-peer learning, quality improvement, emergency medicine residency education, case-based learning

## Abstract

**Introduction:**

We conducted an educational intervention using a peer-to-peer case review process to improve emergency medicine (EM) resident sepsis knowledge and diagnostic confidence. Local quality audits in our academic emergency department (ED) identified poor compliance in addressing adult cases of severe sepsis and septic shock, suggesting an educational gap. We evaluated whether a facilitated, peer-to-peer case review process improved EM resident sepsis knowledge and diagnostic confidence.

**Methods:**

We conducted a single-site, pre-test/post-test educational QI evaluation among senior EM residents. Using routinely audited severe sepsis and septic shock cases that did not meet bundle expectations, residents completed brief, asynchronous peer case reviews with a standardized checklist and provided structured written feedback to the original care teams. Educational impact was evaluated at Kirkpatrick Level 2 (learning) using paired pre–post assessments: a 12-item single-best-answer knowledge test and a 4-point self-reported diagnostic confidence survey. Paired pre/post knowledge scores were compared with the Wilcoxon signed rank test; confidence was summarized descriptively. The institutional review board determined this not human research.

**Results:**

Mean knowledge accuracy improved from 70% (8.36/12) to 85% (10.14/12), an increase of 14.9 percentage points (p = 0.003). The proportion scoring in the 76–100% band increased by 29%, and the number of scores over 90% increased by 53%. Confidence increased for sepsis (71.4% to 78.6%) and remained constant for severe sepsis at 78.6% but decreased for septic shock (92.9% to 85.7%).

**Conclusion:**

A resident-led, asynchronous peer case review process was associated with significantly improved sepsis knowledge and minimal gains in diagnostic confidence for sepsis and severe sepsis. This low-resource approach is feasible for unit-level implementation and may be adaptable to other time-sensitive conditions with complex bundle requirements.

## INTRODUCTION

Early recognition and timely management of sepsis remain challenging in the emergency department (ED), particularly for trainees who must interpret evolving diagnostic criteria while making rapid clinical decisions. Despite ongoing educational efforts, residents often struggle to identify subtle or atypical presentations and to apply guideline-concordant therapies under significant time pressure. These challenges can delay the early recognition and initiation of evidence-based treatments that are essential to improving patient outcomes.^1–5^

In the United States, hospitals participate in the Centers for Medicare and Medicaid (CMS) Hospital Inpatient Quality Reporting Program, which includes the SEP-1 sepsis measure used for national benchmarking and public reporting.^6–8^ Deviations from these bundle elements are frequently identified in local quality audits, suggesting targeted educational gaps that affect both care compliance and resident learning needs.

### Local Problem

Using internal quality improvement (QI) audit abstraction with CMS-aligned criteria, we found frequent deviations from expected sepsis bundle care in our academic ED, affecting nine of 22 severe sepsis or septic shock cases (40.9%). The target benchmark for sepsis management is 75%. The department uses a ≥ 75% compliance threshold as an institutional quality target for CMS-aligned sepsis bundle elements and represents a locally defined performance goal informed by CMS reporting expectations and historical departmental performance. These deviations appeared to reflect, in part, gaps in sepsis recognition and clinical knowledge. Because emergency medicine (EM) residents provide much of the frontline ED care and commonly initiate early sepsis evaluation and treatment, we targeted this group for an educational intervention reinforcing diagnostic definitions, bundle elements, and time-sensitive management.^1,7^

### Aim

The aim of this project was to improve senior emergency medicine residents’ sepsis knowledge and diagnostic confidence through a facilitated peer-to-peer case review of audit-identified sepsis cases, measured using pre-post assessments.

## METHODS

### Study Design

This project was conducted as an educational QI evaluation using a single-site, pre-post design to assess changes in resident sepsis knowledge and self-reported diagnostic confidence following a peer-to-peer case review intervention.

### Setting and Context

This study was conducted in a single academic, adult-only ED with approximately 49,000 annual patient visits. The ED is staffed by 68 full-time and 20 adjunct faculty and serves a three-year EM residency program with approximately 33 residents across post-graduate year (PGY)-1 through PGY-3 levels. Residents practice under attending supervision with progressive autonomy across training years. The study site uses the Epic electronic health record (Epic Systems, Verona, WI) for all clinical documentation and order entry. The department maintains an established sepsis quality audit process, supported by a CMS abstractor, to identify cases with deviations from CMS-aligned bundle expectations.^1^

### Participants

Fifteen of 21 senior residents participated during the study period; 14 completed paired pre- and post-intervention assessments (93.3%). Eligible participants included 10 PGY-3 residents and 5 PGY-2 residents. Because the intervention was designed for all senior trainees and the overall sample size was small, PGY-2 and PGY-3 participants were analyzed together as a single cohort, and no subgroup analysis was performed. Residents who were not eligible (n = 6) were on rotations that precluded QI review activities or were on vacation during the intervention window. All participants had received the same standard institutional sepsis training provided during residency orientation and ongoing clinical practice, and none had completed any additional structured or formal sepsis-focused education in the months preceding the intervention, resulting in generally comparable baseline exposure across PGY-2 and PGY-3 residents.

### Intervention

Using internal QI audits, we identified adult ED cases involving severe sepsis or septic shock with missed or delayed recognition and/or treatment relative to CMS-aligned bundle expectations. Cases were drawn from routinely audited encounters (rather than resident-selected) and assigned to senior residents sequentially on a rotational basis to distribute workload across participants. Resident reviewers completed structured, asynchronous written case reviews using a standardized checklist aligned with local CMS-based sepsis definitions and bundle components, which is provided in full in Appendix 1, between April and July 2024 (Appendix 1).^1,7^ The checklist addressed sepsis time-zero identification and key bundle elements (e.g., lactate measurement, blood cultures, antibiotic timing, fluid resuscitation, and reassessment and/or vasopressor initiation when indicated).^1,7^ Reviewers could consult faculty as needed, although faculty did not routinely review or moderate resident-generated feedback before dissemination.. Each review required approximately 15 to 30 minutes to complete. Seventeen audited cases were reviewed, with each resident reviewing 1–2 cases. After completing each review, the resident reviewer provided structured written feedback to the original care team, highlighting diagnostic decision points, missed opportunities relative to bundle expectations, and concrete learning points. No additional sepsis training occurred during this time.

### Measures

Knowledge assessment: A locally developed, 12-item, single-best-answer questionnaire was administered. Each item included four response options with one correct answer. Knowledge outcomes were calculated as raw scores (0-12 correct) and converted to percentages correct; percentages were used as the primary reporting metric throughout the manuscript to facilitate interpretability, with raw scores reported secondarily when describing score distributions. The instrument assessed five domains based on CMS bundle requirements: (1) systemic inflammatory response syndrome (SIRS) criteria; (2) differentiation between sepsis and severe sepsis; (3) diagnostic criteria and organ dysfunction thresholds for severe sepsis; (4) diagnostic criteria for septic shock; and (5) initial management priorities, including recommended fluid resuscitation volumes, empiric broad-spectrum antibiotic therapy, and organism coverage targets.^1,7^

Confidence survey: Resident self-reported confidence in diagnosing sepsis, severe sepsis, and septic shock was assessed using a 4-point Likert scale (1 = not at all confident to 4 = extremely confident). For analytic purposes, responses were dichotomized: ratings of 3 or 4 were classified as “positive” and ratings of 0 or 1 were classified as “negative.” Dichotomization was used to facilitate interpretation of clinically meaningful confidence versus lack thereof in the context of a small sample size, though we acknowledge that collapsing response categories reduces granularity.

Validity review: Experts in EM and professionals with experience in survey methodology reviewed the instruments for face validity and content coverage. Survey design expertise was consulted during the development of the instrument.

### Analysis

Knowledge outcomes were analyzed in R (version 4.1.3, R Core Team, 2022) using a Wilcoxon signed rank test, comparing matched overall pre- and post-intervention raw scores from the same individuals; this version of R was current and in routine institutional use during the study period (April – July 2024). As an educational QI evaluation, analyses were descriptive and focused on learning outcomes rather than hypothesis testing for generalizable effects. Complete case analysis was used to handle missing data. Confidence outcomes were summarized descriptively as the proportions before and after the intervention. No subgroup analyses were conducted by PGY. No adjustment for multiple comparisons was performed.

### Ethical Considerations

The Oregon Health and Science University Institutional Review Board determined that this educational quality improvement activity did not constitute human subjects research. Although designated as a quality improvement initiative, the project was conducted with the intent to disseminate findings for scholarly and educational purposes.

## RESULTS

**Figure 1.**
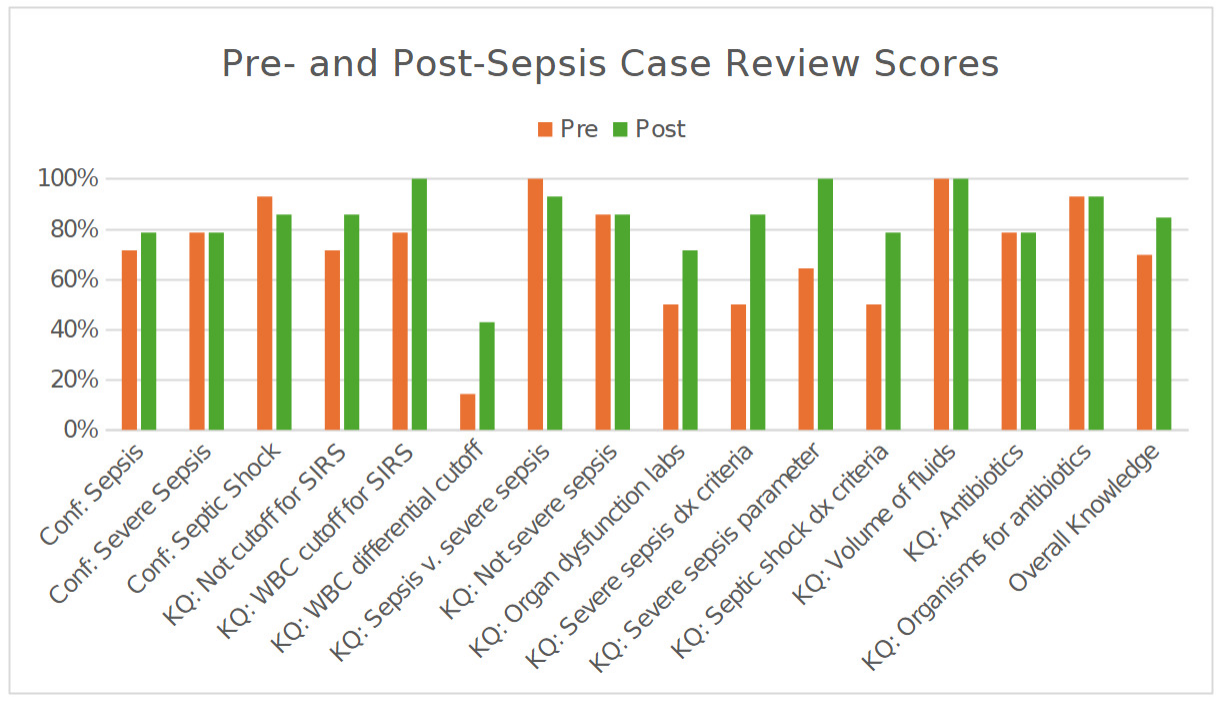
Pre-Post Sepsis Confidence and Knowledge Scores. Percent positive self-reported confidence on diagnosing sepsis and percent correct (out of 12 items) on the sepsis knowledge assessment before and after the intervention. Abbreviations: Conf, confidence; KQ, knowledge question; SIRS, systematic inflammatory response syndrome; WBC, white blood cell.

Fourteen residents completed both surveys. Overall knowledge scores increased from pre- to post-intervention (Figure 1). Overall knowledge significantly improved from 70.0% pre-intervention to 85.0% post-intervention (p = 0.003). On the 12-item assessment, mean scores increased from 8.4 (SD 1.74) to 10.1 (SD 1.41), an average gain of 1.79 items. Post-intervention score distributions showed a higher concentration of scores in the upper accuracy ranges. The proportion of residents scoring in the 76–100% accuracy band increased by 29.0 percentage points, and the proportion scoring at least 90% increased by 53.0 percentage points. Item-level changes showed increases in questions assessing diagnostic criteria and organ dysfunction parameters for severe sepsis. Scores for the white blood cell (WBC) differential item related to SIRS criteria remained lower than other items at both time points.

Self-reported confidence proportions before and after the intervention are presented in Figure 1. The proportion of residents reporting “positive” confidence increased from 71.4% to 78.6% for sepsis diagnosis and remained at 78.6% for severe sepsis. For septic shock, the proportion reporting “positive” confidence changed from 92.9% to 85.7%. Raw scores are reported to one decimal place and percentages to one decimal place throughout to ensure numerical consistency.

## DISCUSSION

In this single-site educational initiative, an asynchronous, resident-led peer case review process was associated with a substantial improvement in sepsis knowledge. The percentage correct increased from 69.6% to 84.5%, an absolute gain of 14.9 percentage points (p = 0.003). The shift toward higher scores suggests that the intervention improved both overall knowledge and mastery of core concepts for a subset of learners. This study employed a case review framework using QI audits as an educational strategy in graduate medical education, integrating established QI processes with clinical teaching and achieving a Kirkpatrick Level 2 (learning) outcome.

Some of the observed improvement in knowledge scores may also reflect a testing effect related to repeated exposure to similar assessment items, although the magnitude of change and item-level patterns suggest learning beyond simple test familiarity.

This intervention incorporated several established educational principles. The use of locally audited cases provided authentic, context-specific learning consistent with case-based learning approaches shown to improve engagement and knowledge acquisition.^9–13^ The standardized checklist clarified diagnostic and management expectations and functioned as a cognitive aid to reduce omission and ambiguity during review.^14,15^ Structured written feedback supported reflection on clinical decision-making and reinforced key learning points.^16–19^ Together, these elements reflect core principles of experiential learning and feedback-informed practice commonly applied in medical education.^20^ Future work is needed to improve knowledge related to WBC differential findings.

Self-reported confidence improved for diagnosing sepsis and remained constant for severe sepsis but declined modestly for septic shock. This modest decrease in septic shock confidence (from 92.9% to 85.7%) may reflect improved diagnostic calibration after reviewing complex or borderline cases, as exposure to nuanced presentations can heighten awareness of uncertainty rather than indicating diminished competence. Future iterations could intentionally oversample shock-focused cases and add explicit prompts to the review checklist regarding reassessment timing, dynamic response to fluids, and escalation decisions (e.g., vasopressor initiation when indicated).

The intervention was brief, asynchronous, integrated into an established audit pipeline, and required minimal additional resources. Reviews were brief, required, integrated into an established audit pipeline, and conducted asynchronously, which minimized the scheduling burden while leveraging existing QI infrastructure.^21,22^ Rotational assignment distributed workload and reduced the potential for selective case choice. Notably, this type of intervention may have the potential to support a learning culture oriented towards improvement. Peer-to-peer review may help frame missed opportunities as shared learning rather than individual failure, which could promote psychological safety and encourage discussion of diagnostic uncertainty.^23–26^ These cultural effects were not directly measured and should be considered hypotheses for future study rather than demonstrated outcomes.

## LIMITATIONS

This work is limited by its single-site design with a small sample size, limiting generalizability. It relied on proximal outcomes (knowledge and self-reported confidence) that may have been impacted by retesting effects and response bias in self-reported confidence measures. Although the magnitude of knowledge gains exceeded what is typically expected from test–retest effects in brief assessments, the absence of a control group means that retesting effects cannot be fully excluded. We did not assess downstream clinical outcomes (e.g., bundle adherence, time to antibiotics) or patient outcomes. The knowledge and confidence instruments were locally developed; although reviewed for face and content validity, they were not formally psychometrically validated. Longer-term durability of learning was not assessed.

Despite these limitations, the intervention provides a low-resource, scalable approach to addressing time-sensitive clinical care education. By combining audit-identified cases, a shared checklist, and structured feedback, peer case review may help align resident mental models with institutional expectations and reduce variability in early sepsis care. Similar approaches may be applicable to other ED priorities requiring rapid recognition and standardized initial management (e.g., stroke, acute coronary syndrome, and trauma).

## CONCLUSIONS

Facilitated, resident peer case review of audit-identified adult ED sepsis cases was associated with significant improvements in sepsis knowledge among senior residents. This low-resource, asynchronous educational model appears feasible for unit-level. Future work should evaluate whether this educational approach translates into improvements in objective clinical metrics such as sepsis bundle compliance, time-to-antibiotics, and other patient-centered outcomes.

## Footnotes

1 The department also participates in broader multicenter quality-improvement initiatives, including the Assessment of Implementation of Methods in Sepsis and Respiratory Failure (AIM-SRF) trial.^27^

## Appendix 1. Emergency Department Sepsis Case Review Checklist

### Section 1. Recognition and Clinical Assessment in the Emergency Department

1. Did the patient meet SIRS criteria in the Emergency Department?
  - Comments:
  - Options: Yes / No / Not applicable
2. Did the patient meet sepsis criteria in the Emergency Department?
  - Comments:
  - Options: Yes / No / Not applicable
3. Did the patient meet severe sepsis (sepsis with acute organ dysfunction) criteria in the Emergency Department?
  - Comments:
  - Options: Yes / No / Not applicable
4. Did the patient meet septic shock criteria in the Emergency Department?
  - Comments:
  - Options: Yes / No / Not applicable

### Section 2. Evaluation of Clinical Management of Severe Sepsis and Septic Shock

1. Was an initial lactate level obtained?
  - Comments:
  - Options: Yes / No / Not applicable
2. Were blood cultures obtained within 3 hours of meeting severe sepsis or septic shock criteria?
  - Comments:
  - Options: Yes / No / Not applicable
3. Did the patient receive appropriate broad-spectrum antibiotics within 3 hours of meeting criteria?
  - Comments:
  - Options: Yes / No / Not applicable
4. Did the patient receive 30 mL/kg crystalloid fluids or have a documented exemption?
  - Comments:
  - Options: Yes / No / Not applicable
5. Did the patient receive vasopressors after fluid resuscitation for septic shock?
  - Comments:
  - Options: Yes / No / Not applicable
6. Was a repeat lactate obtained within 6 hours if initial lactate > 2 mmol/L?
  - Comments:
  - Options: Yes / No / Not applicable

### Section 3. Overall Care Team Assessment

1. Based on the review, did the patient receive guideline-concordant care in the Emergency Department?
  - Comments:
  - Options: Yes / No / Not applicable
